# Supplementary material for: Association Between Temperature, Sunlight Hours, and Daily Steps in School-Aged Children over a 35-Week Period
Source: J Clin Med. 2024 Dec 17;13(24):7679. doi: 10.3390/jcm13247679 (PMC11677288; doi:10.3390/jcm13247679)
Supplement: Supplementary file 1 [file jcm-13-07679-s001.zip › jcm-3244325-supplementary.pdf]

Supplementary material

**Table S1.** STROBE Statement. Checklist of items that should be included in reports of longitudinal studies.	2
**Table S2.** Characteristics of the study areas.	4
**Table S3.** Daily steps, daily sunlight hours, and average daily temperature (ºC), by week of the year and sex.	5
**Table S4.** Multivariable linear regression model of daily steps (total sample, girls, and boys) and daily sunlight hours and average weekly temperature (ºC) for 35 weeks.	7
**Figure. S1.** Diagram flow of the study participants in the current study, from the original e-MOVI project.	8
**Figure S2.** Map of the study area.	9
**Figure S3.** Scatterplots illustrating LOESS regression analysis between weeks of the year (from week 41 2022 to week 23 2023) and daily steps, average daily temperature (ºC), and daily sunlight hours, by sex. Complete n (n girls = 333, n boys = 322): Week 49 to Week 17. Christmas holidays correspond to week 52 and 1. Easter Holidays correspond to week 14.	10
**Figure S4.** Scatterplots illustrating LOESS regression analysis between daily steps and average daily temperature (ºC) and daily sunlight hours, by sex.	11
**Figure S5.** Scatterplots illustrating LOESS regression analysis between average daily temperature (ºC) and daily sunlight hours.	12
**Figure S6.** Interaction between average daily temperature and daily sunlight hours for the mean difference in daily steps.	13
**Figure S7.** Interaction between average daily temperature and daily sunlight hours for the mean difference in daily steps by sex.	14

Table S1. STROBE Statement. Checklist of items that should be included in reports of longitudinal studies.

	Item No	Recommendation	Page No
**Title and abstract**	1	(*a*) Indicate the study's design with a commonly used term in the title or the abstract	1
		(*b*) Provide in the abstract an informative and balanced summary of what was done and what was found	2
**Introduction**			
Background/rationale	2	Explain the scientific background and rationale for the investigation being reported	3
Objectives	3	State specific objectives, including any prespecified hypotheses	3
**Methods**			
Study design	4	Present key elements of study design early in the paper	4
Setting	5	Describe the setting, locations, and relevant dates, including periods of recruitment, exposure, follow-up, and data collection	4
Participants	6	(*a*) Give the eligibility criteria, and the sources and methods of selection of participants	4
Variables	7	Clearly define all outcomes, exposures, predictors, potential confounders, and effect modifiers. Give diagnostic criteria, if applicable	5
Data sources/ measurement	8	For each variable of interest, give sources of data and details of methods of assessment (measurement). Describe comparability of assessment methods if there is more than one group	5, 6
Bias	9	Describe any efforts to address potential sources of bias	6
Study size	10	Explain how the study size was arrived at	4, Figure S1
Quantitative variables	11	Explain how quantitative variables were handled in the analyses. If applicable, describe which groupings were chosen and why	5
Statistical methods	12	(*a*) Describe all statistical methods, including those used to control for confounding	5, 6
		(*b*) Describe any methods used to examine subgroups and interactions	5
		(*c*) Explain how missing data were addressed	6
		(*d*) If applicable, describe analytical methods taking account of sampling strategy	NA
		(*e*) Describe any sensitivity analyses	NA

**Results**			
Participants	13	(a) Report numbers of individuals at each stage of study—eg numbers potentially eligible, examined for eligibility, confirmed eligible, included in the study, completing follow-up, and analysed	4, Figure S1
		(b) Give reasons for non-participation at each stage	4, Figure S1
		(c) Consider use of a flow diagram	Figure S1
Descriptive data	14	(a) Give characteristics of study participants (eg demographic, clinical, social) and information on exposures and potential confounders	1, Table 1, Figure S2, Table S2-S3
		(b) Indicate number of participants with missing data for each variable of interest	4, Figure S1
Outcome data	15	Report numbers of outcome events or summary measures	Table S3
Main results	16	(*a*) Give unadjusted estimates and, if applicable, confounder-adjusted estimates and their precision (eg, 95% confidence interval). Make clear which confounders were adjusted for and why they were included	6, 7, Figures 1-2, Tables 2-3, Table S4
		(*b*) Report category boundaries when continuous variables were categorized	7, Tables 2-3
		(*c*) If relevant, consider translating estimates of relative risk into absolute risk for a meaningful time period	NA
Other analyses	17	Report other analyses done—eg analyses of subgroups and interactions, and sensitivity analyses	8, 9, Tables 2-3, Table S4, Figures S3-S6
**Discussion**			
Key results	18	Summarise key results with reference to study objectives	8
Limitations	19	Discuss limitations of the study, taking into account sources of potential bias or imprecision. Discuss both direction and magnitude of any potential bias	9, 10
Interpretation	20	Give a cautious overall interpretation of results considering objectives, limitations, multiplicity of analyses, results from similar studies, and other relevant evidence	8, 9
Generalisability	21	Discuss the generalisability (external validity) of the study results	10
**Other information**			
Funding	22	Give the source of funding and the role of the funders for the present study and, if applicable, for the original study on which the present article is based	10, 11

Table S2. Characteristics of the study areas.

Site	n	Coordinates	Altitude (m a.s.l.)	Average temperature (ºC)*	Sunlight hours*	Daily steps*	Study period
**Iniesta**	89	39°26′40″N 1°44′55″O	769	10.09 ± 0.00	10.72 ± 0.00	12,109 ± 2,934	Week 41 2022 - Week 16 2023
**Motilla del Palancar**	117	39°33′47″N 1°54′43″O	836	10.15 ± 0.03	10.82 ± 0.07	11,106 ± 3,156	Week 41 2022 - Week 16 2023 (n = 27)<br>Week 42 2022 - Week 17 2023 (n = 57)<br>Week 43 2022 - Week 18 2023 (n = 33)
**San Clemente**	106	39°24′14″N 2°25′46″O	722	10.16 ± 0.03	10.95 ± 0.06	10,996 ± 3,312	Week 43 2022 - Week 18 2023 (n = 78)<br>Week 44 2022 - Week 19 2023 (n = 28)
**Quintanar del Rey**	160	39°20′44″N 1°55′41″O	728	10.11 ± 0.01	11.12 ± 0.07	11,385 ± 3,026	Week 44 2022 - Week 19 2023 (n = 80)<br>Week 45 2022 - Week 20 2023 (n = 80)
**Mota del Cuervo**	84	39°30′01″N 2°52′05″O	714	10.26 ± 0.00	11.34 ± 0.00	11,249 ± 2,832	Week 46 2022 - Week 21 2023
**Las Pedroñeras**	99	39°26′52″N 2°40′18″O	704	10.58 ± 0.15	11.55 ± 0.08	10,945 ± 2,724	Week 47 2022 - Week 22 2023(n = 76)<br>Week 48 2022 - Week 23 2023 (n = 23)
*Values are mean ± standard deviation. Abbreviation: m a.s.l. = metres above sea level							

Table S3. Daily steps, daily sunlight hours, and average daily temperature (ºC), by week of the year and sex.

	Total sample				Girls				Boys			
**Annual week**	***n***	***Daily steps***	***Daily sunlight hours***	***Average daily temperature (ºC)***	n	***Daily steps***	***Daily sunlight hours***	***Average daily temperature (ºC)***	n	***Daily steps***	***Daily sunlight hours***	***Average daily temperature (ºC)***
**Week 41 2022**	116	11163 ± 3565	11.24 ± 0.00	17.29 ± 0.00	62	9493 ± 3180	11.24 ± 0.00	17.29 ± 0.00	54	13080 ± 2988	11.24 ± 0.00	17.29 ± 0.00
**Week 42 2022**	173	11171 ± 3294	10.95 ± 0.00	18.34 ± 0.00	92	9910 ± 3075	10.95 ± 0.00	18.34 ± 0.00	81	12603 ± 2445	10.95 ± 0.00	18.34 ± 0.00
**Week 43 2022**	285	11284 ± 3423	10.67 ± 0.00	18.50 ± 0.00	142	9747 ± 2527	10.67 ± 0.00	18.50 ± 0.00	143	12799 ± 3520	10.67 ± 0.00	18.50 ± 0.00
**Week 44 2022**	392	10890 ± 3640	10.40 ± 0.01	13.41 ± 0.00	197	9344 ± 2915	10.40 ± 0.01	13.41 ± 0.00	195	12451 ± 3638	10.40 ± 0.01	13.41 ± 0.00
**Week 45 2022**	472	10825 ± 3710	10.16 ± 0.01	12.07 ± 0.10	241	9508 ± 3156	10.16 ± 0.01	12.08 ± 0.00	231	12198 ± 3753	10.16 ± 0.01	12.07 ± 0.14
**Week 46 2022**	556	10597 ± 3741	9.93 ± 0.01	9.98 ± 0.05	280	9478 ± 3262	9.93 ± 0.01	9.99 ± 0.00	276	11732 ± 3857	9.93 ± 0.01	9.98 ± 0.07
**Week 47 2022**	632	10679 ± 3830	9.73 ± 0.01	9.13 ± 0.17	319	9383 ± 3076	9.73 ± 0.01	9.14 ± 0.13	313	12001 ± 4068	9.73 ± 0.01	9.13 ± 0.19
**Week 48 2022**	655	10516 ± 3834	9.57 ± 0.01	6.74 ± 0.09	333	9352 ± 3294	9.57 ± 0.01	6.74 ± 0.07	322	11719 ± 3984	9.57 ± 0.01	6.74 ± 0.10
**Week 49 2022**	655	9724 ± 3972	9.45 ± 0.01	8.06 ± 0.14	333	8741 ± 3293	9.45 ± 0.01	8.05 ± 0.12	322	10741 ± 4345	9.45 ± 0.01	8.06 ± 0.17
**Week 50 2022**	655	10458 ± 3843	9.38 ± 0.01	10.18 ± 0.04	333	9393 ± 3355	9.38 ± 0.01	10.18 ± 0.03	322	11560 ± 4007	9.38 ± 0.01	10.18 ± 0.04
**Week 51 2022**	655	11294 ± 4189	9.35 ± 0.01	9.62 ± 0.06	333	9846 ± 3403	9.35 ± 0.01	9.63 ± 0.05	322	12791 ± 4399	9.35 ± 0.01	9.62 ± 0.07
**Week 52 2022**	655	10372 ± 4484	9.38 ± 0.01	8.79 ± 0.10	333	9187 ± 3729	9.39 ± 0.01	8.79 ± 0.08	322	11598 ± 4859	9.38 ± 0.01	8.78 ± 0.11
**Week 1 2023**	655	10099 ± 4235	9.46 ± 0.01	7.38 ± 0.07	333	9029 ± 3486	9.46 ± 0.01	7.38 ± 0.06	322	11206 ± 4643	9.46 ± 0.01	7.38 ± 0.08
**Week 2 2023**	655	10543 ± 3861	9.59 ± 0.01	6.30 ± 0.13	333	9400 ± 3379	9.59 ± 0.01	6.30 ± 0.10	322	11725 ± 3975	9.59 ± 0.01	6.30 ± 0.15
**Week 3 2023**	655	10712 ± 4032	9.76 ± 0.01	4.40 ± 0.12	333	9413 ± 3363	9.76 ± 0.01	4.40 ± 0.10	322	12057 ± 4225	9.76 ± 0.01	4.40 ± 0.14
**Week 4 2023**	655	10864 ± 4073	9.97 ± 0.01	2.64 ± 0.26	333	9667 ± 3535	9.97 ± 0.01	2.63 ± 0.21	322	12101 ± 4224	9.97 ± 0.01	2.64 ± 0.30
**Week 5 2023**	655	11236 ± 4331	10.20 ± 0.00	6.40 ± 0.15	333	10081 ± 3935	10.20 ± 0.00	6.40 ± 0.12	322	12430 ± 4403	10.20 ± 0.00	6.39 ± 0.17
**Week 6 2023**	655	11058 ± 4630	10.46 ± 0.00	4.25 ± 0.20	333	9838 ± 3940	10.46 ± 0.00	4.24 ± 0.16	322	12320 ± 4947	10.46 ± 0.00	4.25 ± 0.23
**Week 7 2023**	655	11731 ± 4752	10.73 ± 0.00	7.17 ± 0.02	333	10434 ± 4254	10.73 ± 0.00	7.17 ± 0.01	322	13073 ± 4871	10.73 ± 0.00	7.17 ± 0.02
**Week 8 2023**	655	11483 ± 4592	11.02 ± 0.00	6.88 ± 0.33	333	10296 ± 4339	11.02 ± 0.00	6.89 ± 0.26	322	12710 ± 4530	11.02 ± 0.00	6.87 ± 0.38
**Week 9 2023**	655	11382 ± 4379	11.31 ± 0.00	2.13 ± 0.66	333	10287 ± 4242	11.31 ± 0.00	2.12 ± 0.54	322	12515 ± 4234	11.31 ± 0.00	2.15 ± 0.77
**Week 10 2023**	655	11599 ± 4450	11.61 ± 0.00	11.90 ± 0.01	333	10574 ± 4363	11.61 ± 0.00	11.90 ± 0.01	322	12659 ± 4295	11.61 ± 0.00	11.90 ± 0.02

**Week 11 2023**	655	11844 ± 4539	11.91 ± 0.00	12.11 ± 0.07	333	10925 ± 4652	11.91 ± 0.00	12.11 ± 0.06	322	12796 ± 4219	11.91 ± 0.00	12.11 ± 0.09
**Week 12 2023**	655	11940 ± 4723	12.21 ± 0.00	13.20 ± 0.02	333	10948 ± 4785	12.21 ± 0.00	13.20 ± 0.02	322	12965 ± 4437	12.21 ± 0.00	13.20 ± 0.02
**Week 13 2023**	655	12447 ± 4901	12.51 ± 0.00	13.48 ± 0.03	333	11570 ± 4941	12.51 ± 0.00	13.48 ± 0.03	322	13353 ± 4697	12.51 ± 0.00	13.48 ± 0.04
**Week 14 2023**	655	12301 ± 5097	12.81 ± 0.00	13.03 ± 0.10	333	11603 ± 5145	12.81 ± 0.00	13.03 ± 0.08	322	13022 ± 4951	12.81 ± 0.00	13.03 ± 0.11
**Week 15 2023**	655	12416 ± 4820	13.11 ± 0.00	14.47 ± 0.02	333	11647 ± 4872	13.11 ± 0.00	14.47 ± 0.02	322	13210 ± 4640	13.11 ± 0.00	14.47 ± 0.02
**Week 16 2023**	655	12400 ± 4809	13.39 ± 0.00	14.76 ± 0.32	333	11605 ± 4889	13.39 ± 0.00	14.76 ± 0.26	322	13222 ± 4590	13.39 ± 0.00	14.77 ± 0.37
**Week 17 2023**	539	12072 ± 4146	13.66 ± 0.00	19.48 ± 0.07	271	1113 5 ± 3913	13.66 ± 0.00	19.48 ± 0.06	268	13019 ± 4166	13.66 ± 0.00	19.48 ± 0.08
**Week 18 2023**	482	11896 ± 4053	13.92 ± 0.01	18.56 ± 0.16	241	10839 ± 3657	13.92 ± 0.01	18.56 ± 0.13	241	12953 ± 4160	13.92 ± 0.01	18.55 ± 0.18
**Week 19 2023**	371	12077 ± 4017	14.16 ± 0.01	16.53 ± 0.25	192	11115 ± 3791	14.16 ± 0.01	16.54 ± 0.20	179	13109 ± 4006	14.16 ± 0.01	16.52 ± 0.30
**Week 20 2023**	263	12412 ± 4245	14.38 ± 0.01	13.74 ± 0.26	136	11921 ± 4218	14.38 ± 0.01	13.73 ± 0.21	127	12939 ± 4227	14.38 ± 0.01	13.75 ± 0.31
**Week 21 2023**	183	11782 ± 3663	14.57 ± 0.00	16.17 ± 0.02	92	11131 ± 3874	14.57 ± 0.00	16.17 ± 0.02	91	12439 ± 3330	14.57 ± 0.00	16.17 ± 0.02
**Week 22 2023**	99	11521 ± 3348	14.71 ± 0.00	16.40 ± 0.26	53	11112 ± 3526	14.71 ± 0.00	16.43 ± 0.36	46	11992 ± 3102	14.71 ± 0.00	16.38 ± 0.00
**Week 23 2023**	23	11566 ± 2910	14.82 ± 0.00	18.99 ± 0.00	14	10427 ± 2561	14.82 ± 0.00	18.99 ± 0.00	9	13338 ± 2613	14.82 ± 0.00	18.99 ± 0.00
Values are mean ± standard deviation.												

Table S4. Multivariable linear regression model of daily steps (total sample, girls, and boys) and daily sunlight hours and average weekly temperature (ºC) for 35 weeks.

		**Daily sunlight hours**		**Average daily temperature**	
		β ± SD	p-value	β ± SD	p-value
**Total sample**	M_0	315 ± 237	**< 0.001**	74 ± 130	**0.002**
	M_1	335 ± 331	**< 0.001**	-10 ± 124	0.617
**Girls**	M_0	416 ± 260	**< 0.001**	86 ± 166	**0.004**
	M_1	484 ± 343	**< 0.001**	-37 ± 124	0.097
**Boys**	M_0	235 ± 278	**< 0.001**	69 ± 112	**< 0.001**
	M_1	192 ± 379	**0.005**	21 ± 142	0.389

Data are presented as beta coefficient (β) ± standard deviation (SD). The values in bold indicate statistical significance at $p < 0.05$. Model 0 (M0): raw data analysis. Model 1 (M1): controlling for average weekly temperature (ºC) or daily sunlight hours.

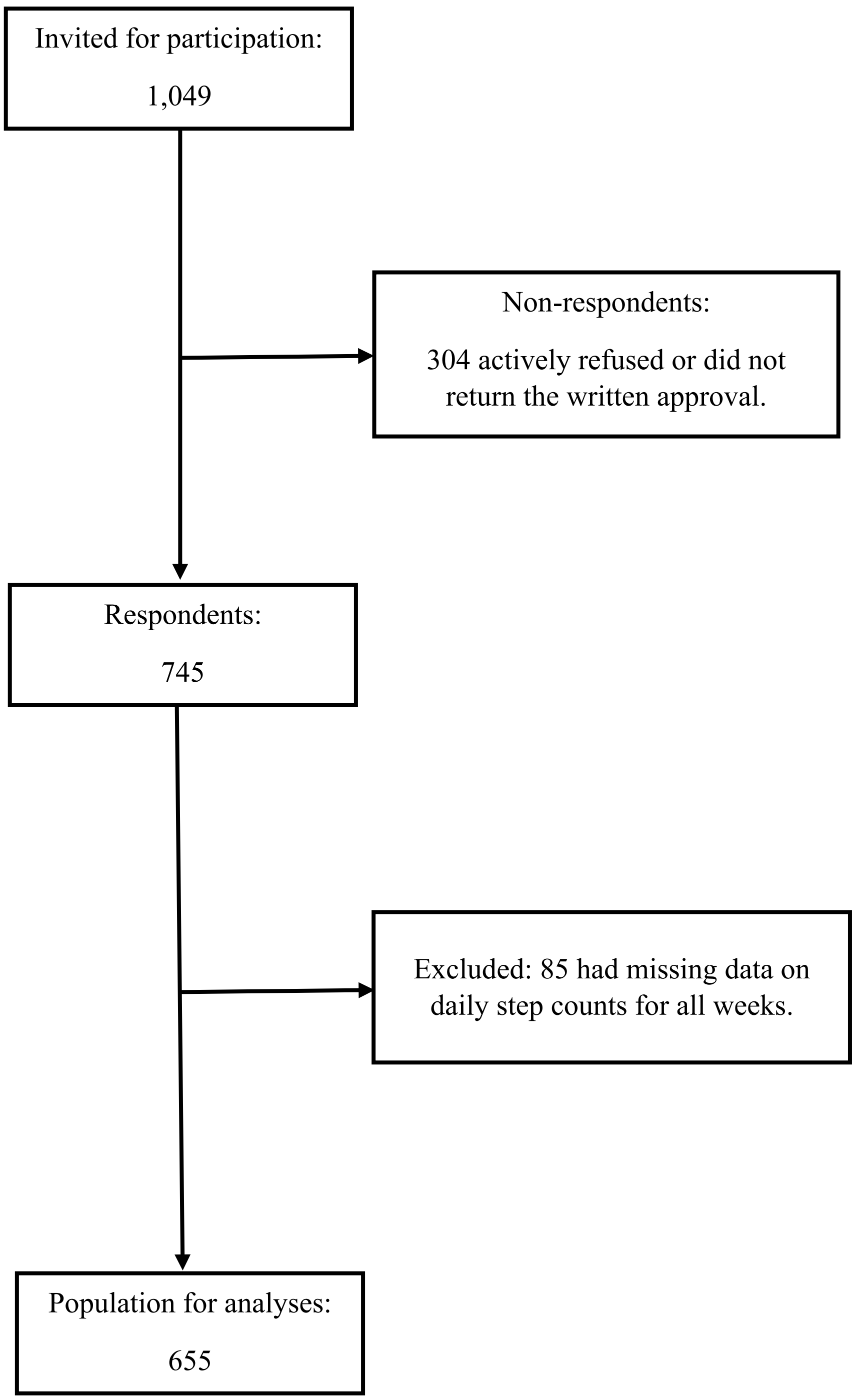

Figure. S1. Diagram flow of the study participants in the current study, from the original e-MOVI project.

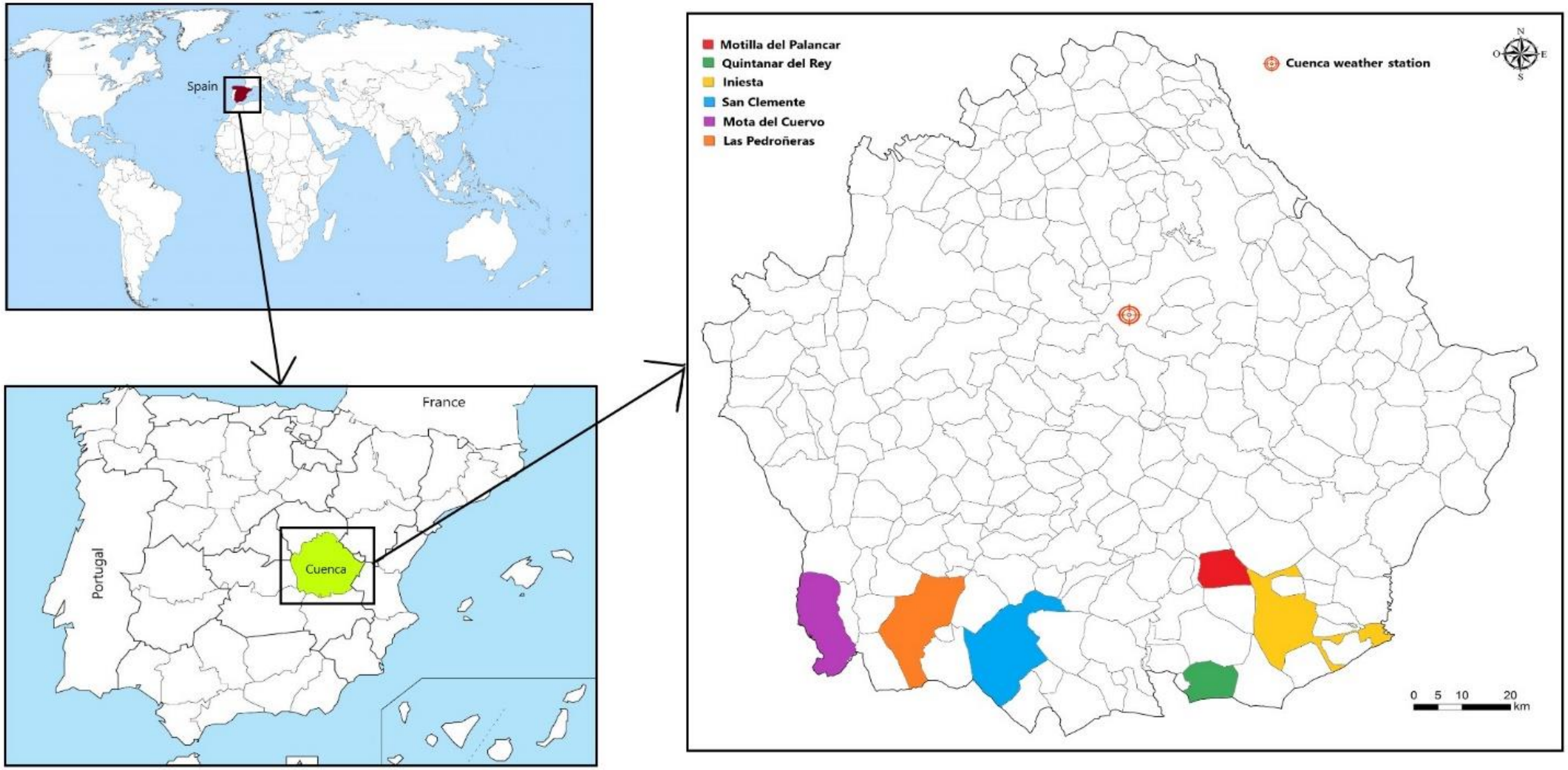

Figure S2. Map of the study area.

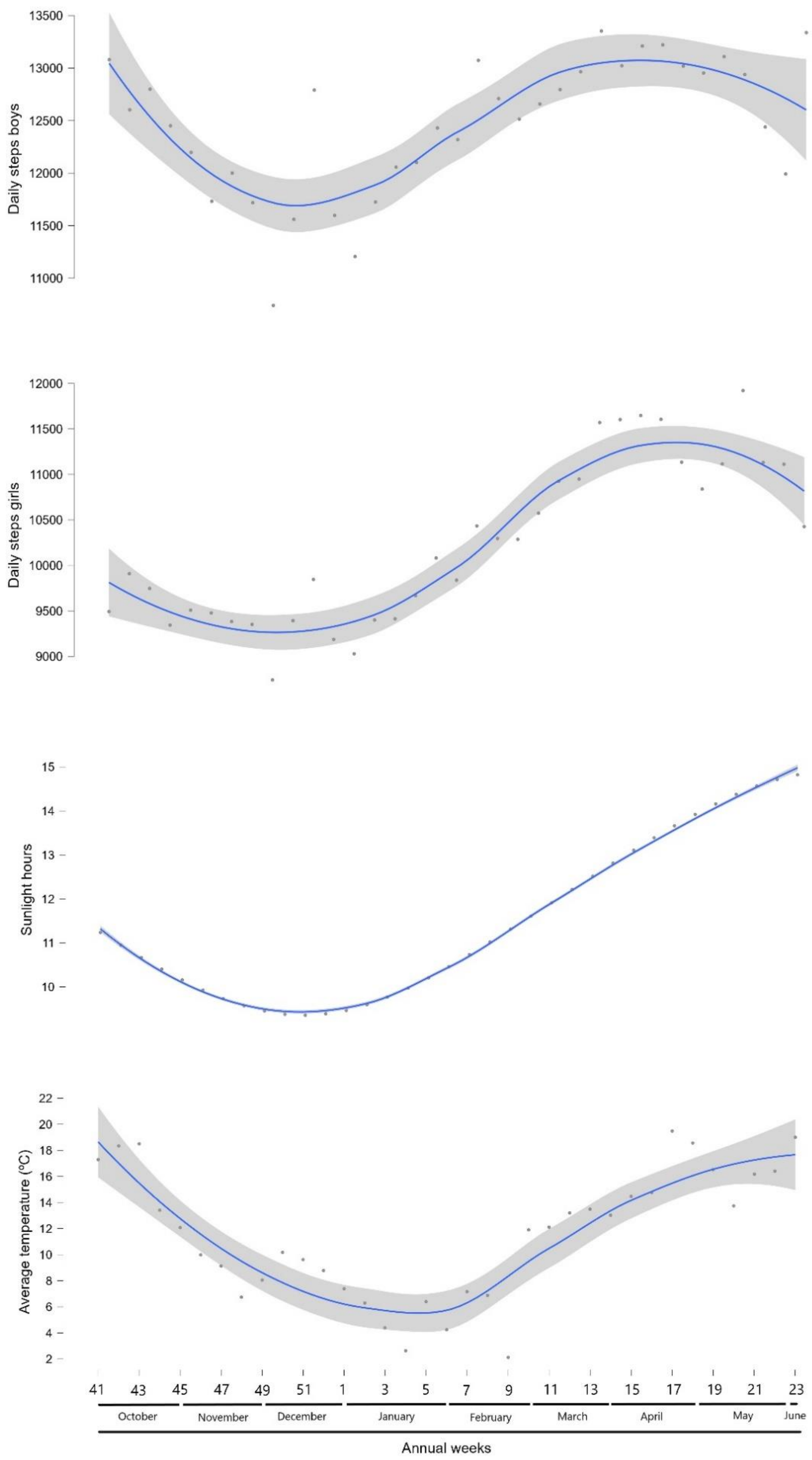

Figure S3. Scatterplots illustrating LOESS regression analysis between weeks of the year (from week 41 2022 to week 23 2023) and daily steps, average daily temperature (ºC), and daily sunlight hours, by sex. Complete n (n girls = 333, n boys = 322): Week 49 to Week 17. Christmas holidays correspond to week 52 and 1. Easter Holidays correspond to week 14.

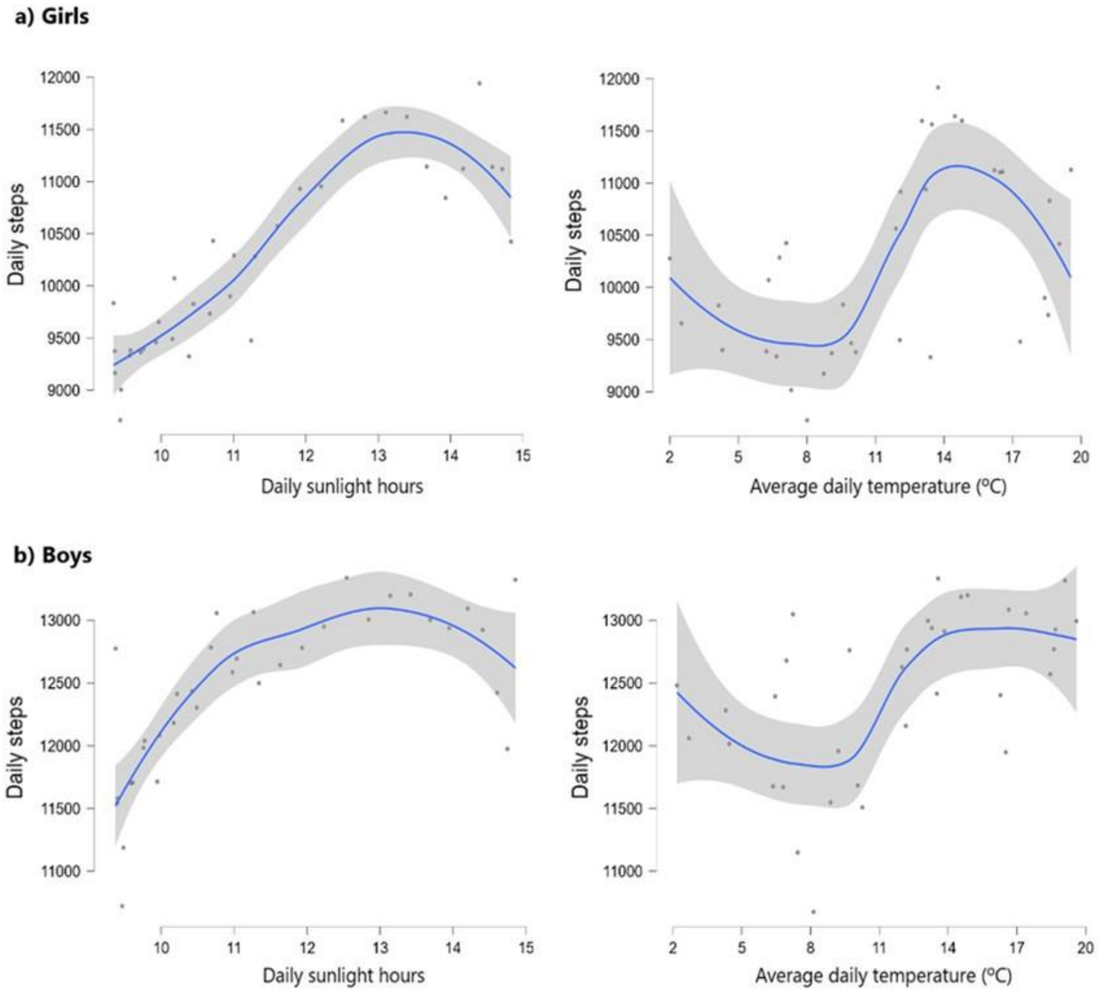

Figure S4. Scatterplots illustrating LOESS regression analysis between daily steps and average daily temperature (ºC) and daily sunlight hours, by sex.

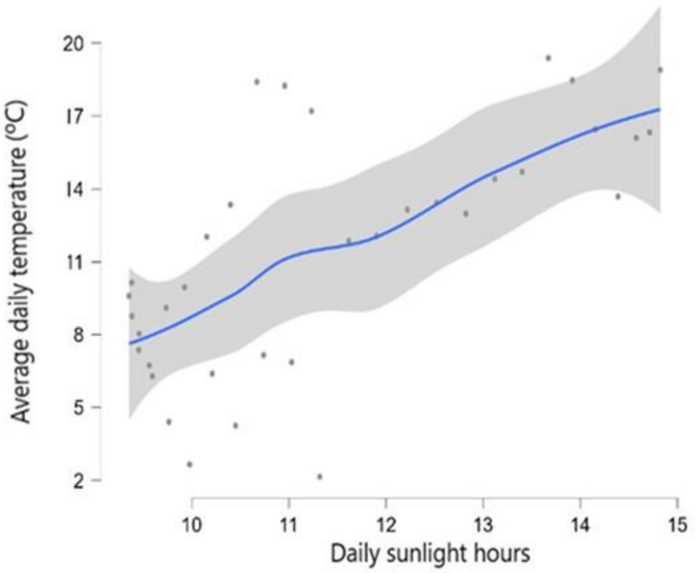

Figure S5. Scatterplots illustrating LOESS regression analysis between average daily temperature (ºC) and daily sunlight hours.

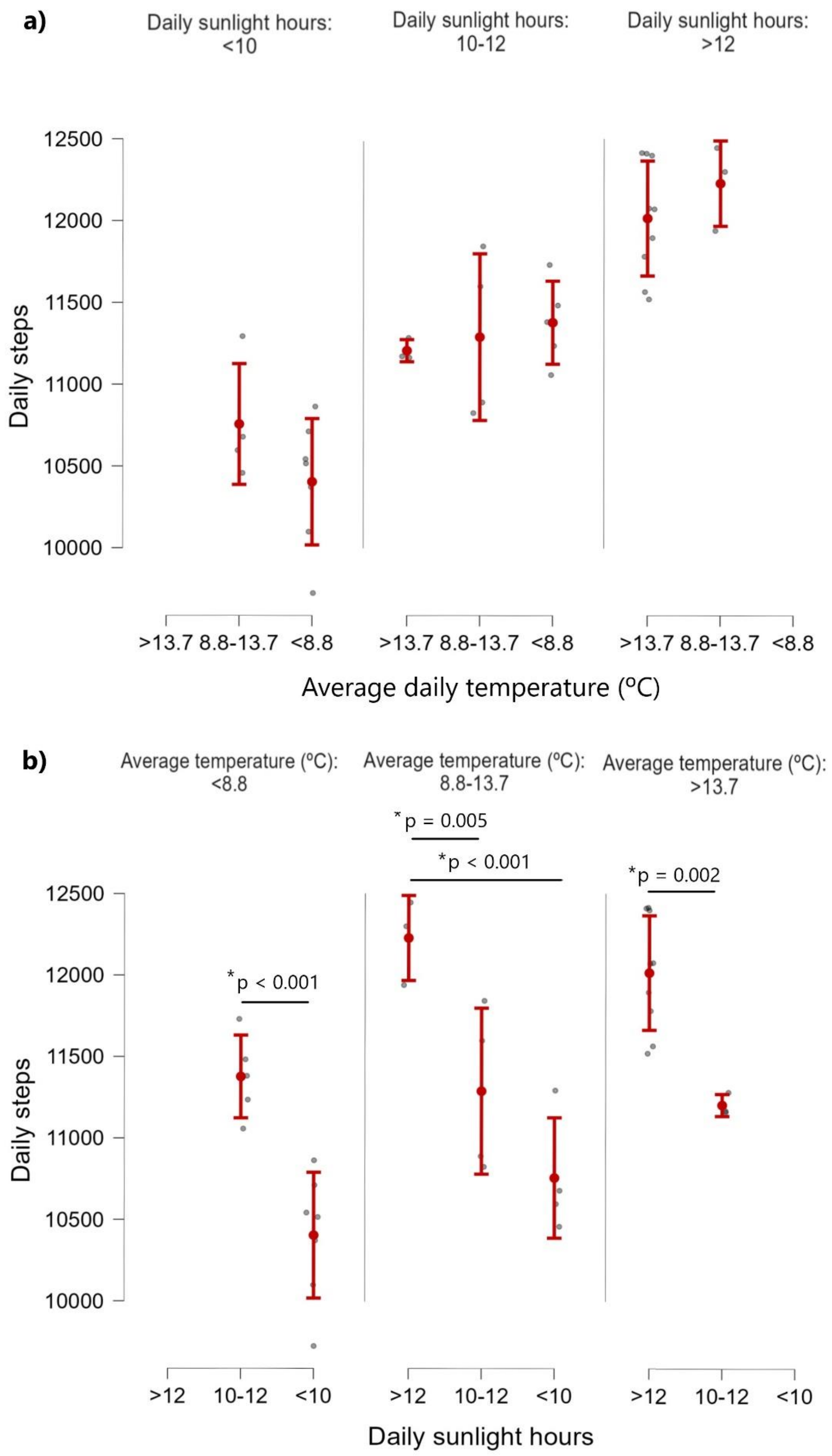

Figure S6. Interaction between average daily temperature and daily sunlight hours for the mean difference in daily steps. * Indicates $p < 0.05$.

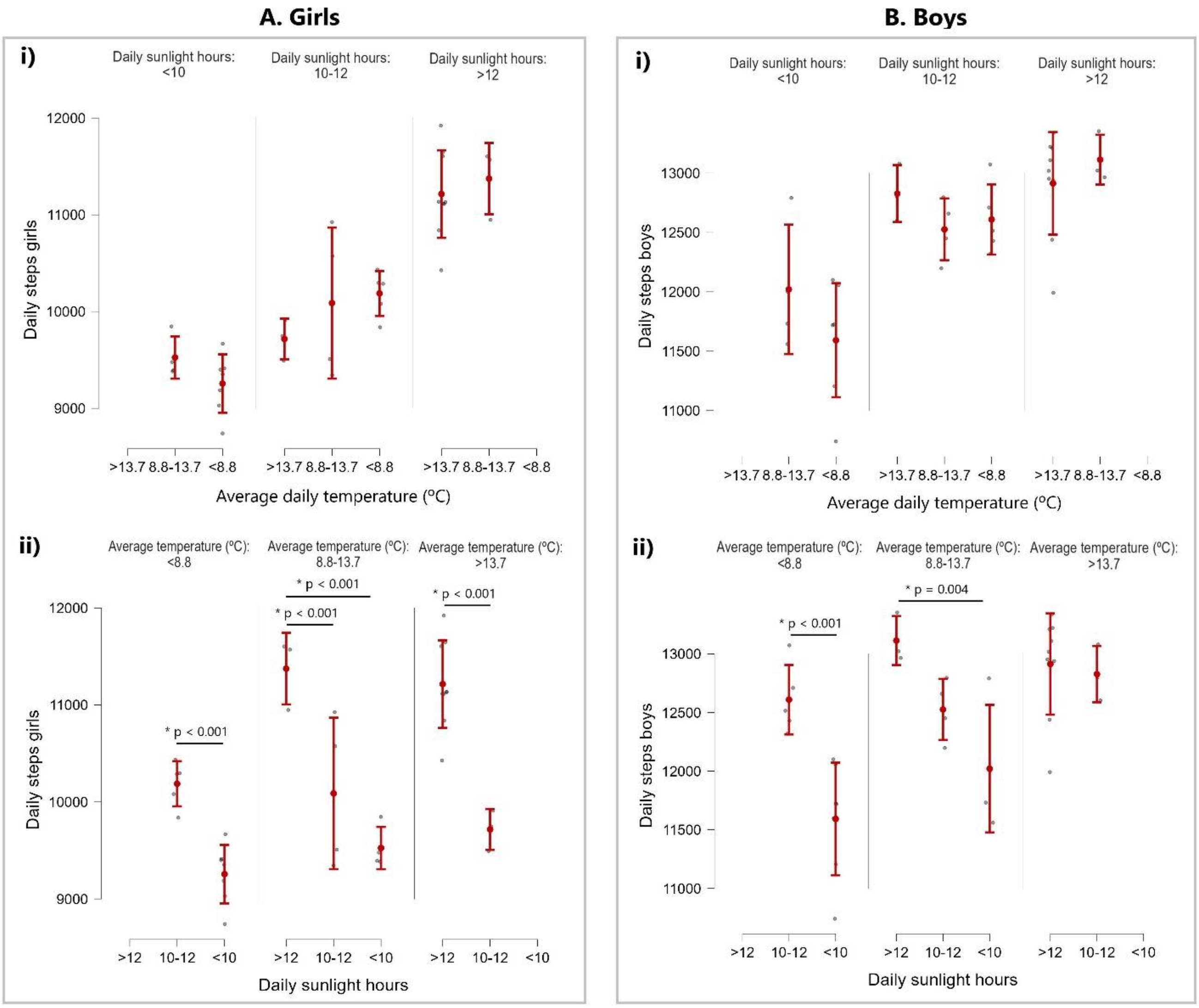

Figure S7. Interaction between average daily temperature and daily sunlight hours for the mean difference in daily steps by sex.
